# Correction: Can we use it? On the utility of *de novo* and reference-based assembly of Nanopore data for plant plastome sequencing

**DOI:** 10.1371/journal.pone.0232195

**Published:** 2020-04-17

**Authors:** The PLOS ONE Staff

## Notice of Republication

An incorrect version of the Data Availability statement was published in error. This article was republished on April 1, 2019, to correct for this error. Please download this article again to view the correct version.
