# Supplementary material for: Can we use it? On the utility of de novo and reference-based assembly of Nanopore data for plant plastome sequencing
Source: PLoS One. 2020 Mar 24;15(3):e0226234. doi: 10.1371/journal.pone.0226234 (PMC7092973; doi:10.1371/journal.pone.0226234)
Supplement: S1 File — (DOCX) [file pone.0226234.s001.docx]

**Tools and commands used for analysis of Illumina and Nanopore sequence data**

**A. Illumina - Read trimming and filtering**

1) Demultiplexing of paired-end reads from a single run

Tool: bcl2fastq

**bcl2fastq -i [/path/to/Runfolder/]Data/Intensities/Basecalls -R [/path/to/Runfolder/] -o [/path/to/Runfolder/]Data/Intensities/Basecalls --sample-sheet [/path/to/Runfolder/]SampleSheet.csv -r 4 -p 8 -w 4 --barcode-mismatches 1 ‑‑ignore-missing-bcls --ignore-missing-filter --ignore-missing-positions --fastq-compression-level 9**

2) Merging reads from different runs

note: keep mate 1 and mate 2 separate!

**cat [reads_mate_1_run_1].fastq [reads_mate_1_run_2].fastq > [mate_1_reads_combined].fastq**

**cat [reads_mate_2_run_1].fastq [reads_mate_2_run_2].fastq > [mate_2_reads_combined].fastq**

3) Adapter and barcode trimming of paired-end reads

Tool: BBDuk (BBTools software suite)

**./bbduk.sh in1=[reads_mate_1].fastq in2=[reads_mate_2].fastq out1=[adapter-trimmed_reads_mate_1].fastq out2=[adapter-trimmed_reads_mate_2].fastq ref=[adapter_sequences].fa ktrim=r k=23 mink=11 hdist=1 tpe tbo**

4) PhiX spike-in removal

Tool: BBDuk (BBTools software suite)

**./bbduk.sh in1=[reads_mate_1].fastq in2=[reads_mate_2].fastq out1=[reads_mate_1].fastq out2=[reads_mate_2].fastq outm=[phix_reads].fastq ref=phix.fa k=31 hdist=1 stats=[statsfile].txt**

5) Primer trimming of paired-end reads

Tool: BBDuk (BBTools software suite)

note: trimming at 5' and 3' ends is done sequentially. Prepare respective primer reference file to only include primers of equal length; if primers have different lengths, prepare several reference files and run command multiple times, using the output files of one run as input for the next

5' trimming:

**./bbduk.sh in1=[reads_mate_1].fastq in2=[reads_mate_2].fastq out1=[5'primertrimmed_reads_1].fastq out2=[5'primertrimmed_reads_2].fastq ref=[primer_length_x_reference_5'end].fasta copyundefined k=[primer_length_x] hdist=1 ktrim=l restrictleft=[primer_length_x] tbo mm=f rcomp=f**

3' trimming:

note: trim primer sequences in the primer reference files to their first 11 bp for this step, but do not combine reference files; run command multiple times as in 5' trimming

**./bbduk.sh in1=[5'primertrimmed_reads_1].fastq in2=[5'primertrimmed_reads_2].fastq out1=[primertrimmed_reads_1].fastq out2=[primertrimmed_reads_2].fastq ref=[primer_length_x_reference_3'end, primers trimmed to the first 11 bp].fasta copyundefined k=11 hdist=0 ktrim=r restrictright=[original_primer_length_x] tbo mm=f rcomp=f**

6) Quality and Length filtering

Tool: BBDuk (BBTools software suite)

**./bbduk.sh in1=[primertrimmed_reads_1].fastq in2=[primertrimmed_reads_2].fastq out1=[processed_reads_1].fastq out2=[processed_reads_2].fastq qtrim=rl trimq=[desired_phred_score, e.g. 20; regions with average quality below this will be trimmed from reads] minlen=50**

**B. Illumina - Reference-based mapping assembly**

1) Mapping reads to reference *L. virgatum*

Tool: BBMap (BBTools software suite)

note: the sequence from the Illumina *de novo* assembly of *L. virgatum* is used as reference

**./bbmap.sh ref=[reference_L_virgatum_without_IRa].fasta nodisk=t in1=[processed_reads_1].fastq in2=[processed_reads_2].fastq out=[mapped_reads].sam vslow=t maxindel=300 threads=[desired_no_of_threads] ambiguous=random killbadpairs=t pairedonly=t pairlen=180 mappedonly=t showprogress2=10 mdtag=t nhtag=t nmtag=t stoptag=t lengthtag=t idtag=t inserttag=t boundstag=t scafstats=[scafstats].txt sortscafs=t qhist=[qhist].txt lhist=[lhist].txt ihist=[ihist].txt ehist=[ehist].txt indelhist=[indelhist].txt statsfile=[stats].txt covstats=[covstats].txt basecov=[basecov].txt secondarycov=f**

2) Mapping reads to reference *Artemisia frigida*

Tool: BBMap (BBTools software suite)

note: the chloroplast genome sequence of *A. frigida* (acc.no. NC_020607.1 in Genbank) is used as reference

**./bbmap.sh ref=[reference_A_frigida_without_IRa].fasta nodisk=t in1=[processed_reads_1].fastq in2=[processed_reads_2].fastq out=[mapped_reads].sam vslow=t maxindel=1500 threads=[desired_no_of_threads] ambiguous=random killbadpairs=t pairedonly=t pairlen=180 mappedonly=t showprogress2=10 mdtag=t nhtag=t nmtag=t stoptag=t lengthtag=t idtag=t inserttag=t boundstag=t scafstats=[scafstats].txt sortscafs=t qhist=[qhist].txt lhist=[lhist].txt ihist=[ihist].txt ehist=[ehist].txt indelhist=[indelhist].txt statsfile=[stats].txt covstats=[covstats].txt basecov=[basecov].txt secondarycov=f**

3) Convert (.sam to .bam), sort and index mappings

Tool: SAMtools

**samtools view -b -h -o [mapped_reads].bam [mapped_reads].sam**

**samtools sort -o [mapping_sorted].bam [mapped reads].bam**

**samtools index -b [mapping_sorted].bam [mapping_sorted].bai**

4) Remove duplicate reads from L. virgatum and Artemisia mappings

Tool: Picard tools

**picard MarkDuplicates Remove_Duplicates=true I=[mapping_sorted].bam O=[mapping_sorted_nodup].bam M=[log].txt**

5) Variant calling

Tool: *callvariants2.sh* (BBTools software suite)

**./callvariants2.sh in=[mapping_sorted_nodup].bam ref=[reference_used_for_mapping].fasta vcf=[variant_output].vcf shist=[stats].txt ploidy=1 rarity=0.51 border=0 qtrim=f trimq=0 realign=f minreadmapq=1 minallelefraction=0.51**

6) Compress and index variant files

Tool: HTSlib

**bgzip [variant_output].vcf**

**tabix -p vcf [variant_output].vcf.gz**

7) Calculate variant statistics

Tool: VCFtools

**vcftools --gzvcf [compressed_and_indexed_variant_file].vcf.gz --out [output_name] ‑‑[option]**

note: exemplary options are e.g. --depth (mean depth per individual), --site-quality (per-site SNP quality), --SNPdensity 1000 (number and density of SNPs in bins of size 1000), --site-mean-depth (mean depth per site averaged across all individuals)

8) Create updated consensus

Tool: BCFtools

**bcftools consensus -f [reference_used_for_mapping].fasta -M N -o [output_consensus].fasta [compressed_and_indexed_variant_file].vcf.gz**

**C. Illumina - *De novo* assembly**

1) *De novo* assembly

Tool: Unicycler

**unicycler -1 [processed_reads_1].fastq -2 [processed_reads_2].fastq -o [path/to/output/folder] –mode conservative**

2) Obtain read coverage of *de novo* assembly via mapping input reads

Tool: BBMap (BBTools software suite)

note: map the reads used for assembly by Unicycler, i.e., the processed reads

**./bbmap.sh ref=[de_novo_assembled_fasta_without_IRa].fasta nodisk=t in1=[processed_reads_1].fastq in2=[processed_reads_2].fastq out=[coveragemapping].sam vslow=t maxindel=100 threads=[desired_no_of_threads] ambiguous=random killbadpairs=t pairedonly=t pairlen=180 mappedonly=t scafstats=[scafstats].txt sortscafs=t statsfile=[stats].txt covstats=[covstats].txt basecov=[basecov].txt secondarycov=f**

**D. Nanopore - Read trimming, filtering and improvement**

1) Basecalling and demultiplexing

Tool: Albacore

note: Use the complete fast5-folder as input, including fail-, pass- and skip-subfolder if present!

**./read_fast5_basecaller.py -i [path/to/input/folder/] -s [path/to/output/folder/] -o [output_format; e.g. fast5,fastq] -f [used_flowcell, e.g. FLO-MIN106] -k [used_kit, e.g. SQK-LSK108] -r --barcoding --disable_pings -t [desired_no_of_threads]**

2) Demultiplexing and adapter / barcode trimming

Tool: Porechop

**./porechop-runner.py -i [path/to/Albacore/pass/folder] --format fastq -b [path/to/folder/for/barcoded/fastqs] --discard_middle --middle_threshold 75 ‑‑end_threshold 75 --end_size 150 -v 2 -t [desired_no_of_threads]**

3) Primer trimming

Tool: BBDuk (BBTools software suite)

note: trimming at 5' and 3' ends is done sequentially as for Illumina. Prepare respective primer reference file with primers in normal, complement, reverse, and reverse-complement orientations; separate reference files for different primer lengths are not needed

5' trimming:

**./bbduk.sh in=[reads].fastq out=[5'primertrimmed_reads].fastq ref=[primer_reference_5'end].fasta k=11 hdist=0 edist=2 mm=f rcomp=f mkf=0.51 threads=[desired_no_of_threads] ktrim=l restrictleft=100 copyundefined refstats=[statsfile].txt**

3' trimming:

**./bbduk.sh in=[5'primertrimmed_reads].fastq out=[primertrimmed_reads].fastq ref=[primer_reference_3'end].fasta k=11 hdist=0 edist=2 mm=f rcomp=f mkf=0.51 threads=[desired_no_of_threads] ktrim=r restrictright=100 copyundefined refstats=[statsfile].txt**

4) Quality and Length filtering

Tool: NanoFilt

**NanoFilt --maxlength 12600 -q [minimum average read quality score; e.g. 7] -s [path/to/Albacore/]sequencing_summary.txt --readtype 1D --logfile [logfile].txt < [primertrimmed_reads].fastq > [processed_reads].fastq**

5) Filter contaminant sequences (from other organisms)

Tool: BLAST+, scripts *fastq2fasta.sh* and *filter_new.sh* (written by Ulrich
 Lautenschlager, Regensburg, available upon request)

note: convert .fastq to .fasta first, then create a BLAST database to BLAST the sequences against (here: from the *A. frigida* chloroplast, available in Genbank at acc.no. NC_020607.1, with the IR_A_ removed), then do the BLAST search, then submit the result to the *filter_new.sh* script by entering the file name under "blast_archives" and setting the cutoff to 70 within the script. Several .fasta files are output by the script; use [processed_reads]_qcov-filtered.fasta to continue

**./fastq2fasta.sh [processed_reads].fastq**

**makeblastdb -in [Genbank_reference].fasta -logfile stderr -input_type fasta -dbtype nucl -out [name_of_database] -title "[title_of_database]"**

**blastn -task megablast -query [processed_reads].fasta -db [name_of_database] -out [output_filename].txt -outfmt 11**

**./filter_new.sh**

6) Split chimeric reads and subsequently remove very short sequences

Tool: Pacasus, GenomeTools, script *long_seq.py* (available at

http://seqanswers.com/forums/showthread.php?t=31046)

note: command refers to Pacasus used with an NVIDIA GPU using CUDA. Change settings to use other supported platforms. Make sure you have the required environment variables correctly set on your system. Setting them might be necessary for each new session; after running Pacasus, optionally check the sequence length distribution with GenomeTools, then remove sequences < 50 bp with *long_seq.py*

**python pacasus.py --logfile=[Pacasus_log].txt --loglevel=WARNING -o [processed_reads_qcov-filtered_dechim].fasta -1 fasta --device_type=GPU ‑‑minimum_read_length=0 --filter_factor=0.0001 --query_coverage=0.001 ‑‑query_identity=0.001 --relative_score=0.001 --base_score=0.50 [processed_reads_qcov-filtered].fasta**

**./gt seqstat -v yes -distlen yes -b 100 [processed_reads_qcov-filtered_dechim].fasta**

**python long_seq.py [processed_reads_qcov-filtered_dechim].fasta [fully_processed_reads].fasta 49**

7) Improve reads for hybrid assembly using Nanocorr

Tool: Nanocorr, script *fastq2fasta.sh* (written by Ulrich Lautenschlager, Regensburg, available upon request)

note: input for Nanocorr are the fully processed Nanopore reads and the processed Illumina reads, both in .fasta format (concatenate forward and reverse Illumina reads into one file). Nanopore reads are partitioned first into 1000 reads per file and 15 files per subfolder, which will be found in the nanocorr folder; navigate into each subfolder and execute the Nanocorr command lines. The result will be 15 *.blast6.r.fa files in every subfolder which can be combined into one [Nanopore_fully_processed_reads_improved].fasta using cat

**cat [processed_reads_1].fastq =[processed_reads_2].fastq > [Illumina_processed_reads].fastq**

**./fastq2fasta.sh [Illumina_processed_reads].fastq [Illumina_processed_reads].fasta**

**python partition.py 1000 15 [Nanopore_fully_processed_reads].fasta**

**$>for j in {1..15}; do**

**echo "SGE_TASK_ID=$j TMPDIR=/tmp [/path/to/Nanocorr/]nanocorr.py [Illumina_processed_reads].fasta [Nanopore_fully_processed_reads].fasta";**

**done | parallel -j 15**

X) Read mapping

Tool: Minimap2, SAMtools

note: reads can be mapped to a known reference at any point of the workflow for quality checking or simply eyeballing using Minimap2 and then SAMtools for preparation for illustration in IGV. The number of unmapped, secondary, supplementary etc. reads is easily summarized with SAMtools *view* (use -f with options 4 (or 4, 8 and 12 when mapping Illumina reads), 0x0100, and 0x0800, respectively)

**./minimap2 -ax map-ont -t [desired_no_of_threads] [reference].fasta [input_reads].fasta/.fastq > [mapped_reads].sam**

**samtools view -b -h -o [mapped_reads].bam [mapped_reads].sam**

**samtools sort -o [mapping_sorted].bam [mapped reads].bam**

**samtools index -b [mapping_sorted].bam [mapping_sorted].bai**

**samtools view -c -f [desired_category_of_reads] [mapped_reads].sam**

**E. Nanopore - Reference-based mapping assembly**

1) Reduce Nanopore read dataset to include only reads ≥ 4200 bp

Tool: NanoFilt

note: use processed reads for reference-based assembly

**NanoFilt -l 4200 -s [path/to/Albacore/]sequencing_summary.txt --readtype 1D ‑‑logfile [logfile].txt < [processed_reads].fastq > [processed_reads_4200].fastq**

2) Mapping reads to reference *L. virgatum*

Tool: *mapPacBio.sh* (BBTools software suite)

note: the sequence from the Illumina *de novo* assembly of *L. virgatum* is used as reference

**./mapPacBio.sh ref=[reference_L_virgatum_without_IRa].fasta nodisk=t in=[processed_reads_4200].fastq out=[mapped_reads].sam maxlen=3000 vslow=t maxindel=300 threads=[desired_no_of_threads] ambiguous=random mappedonly=t showprogress2=10 mdtag=t nhtag=t nmtag=t stoptag=t lengthtag=t idtag=t inserttag=t scafstats=[scafstats].txt sortscafs=t qhist=[qhist].txt lhist=[lhist].txt ehist=[ehist].txt indelhist=[indelhist].txt statsfile=[stats].txt covstats=[covstats].txt basecov=[basecov].txt secondarycov=f**

3) Mapping reads to reference *Artemisia frigida*

Tool: *mapPacBio.sh* (BBTools software suite)

note: the chloroplast genome sequence of *A. frigida* (acc.no. NC_020607.1 in Genbank) is used as reference

**./mapPacBio.sh ref=[reference_A_frigida_without_IRa].fasta nodisk=t in=[processed_reads_4200].fastq out=[mapped_reads].sam maxlen=3000 vslow=t maxindel=1500 threads=[desired_no_of_threads] ambiguous=random mappedonly=t showprogress2=10 mdtag=t nhtag=t nmtag=t stoptag=t lengthtag=t idtag=t inserttag=t scafstats=[scafstats].txt sortscafs=t qhist=[qhist].txt lhist=[lhist].txt ehist=[ehist].txt indelhist=[indelhist].txt statsfile=[stats].txt covstats=[covstats].txt basecov=[basecov].txt secondarycov=f**

4) Convert (.sam to .bam), sort and index mappings (as for Illumina)

Tool: SAMtools

**samtools view -b -h -o [mapped_reads].bam [mapped_reads].sam**

**samtools sort -o [mapping_sorted].bam [mapped reads].bam**

**samtools index -b [mapping_sorted].bam [mapping_sorted].bai**

note: do not deduplicate reads after this step (as done with Illumina reads)!

5) Variant calling

Tool: *callvariants2.sh* (BBTools software suite)

note: check the resulting .vcf files for multiallelic sites (i.e., sites with more than one variant). The variant with the lower quality can be deleted (haploid dataset)

**./callvariants2.sh in=[mapping_sorted].bam ref=[reference_used_for_mapping].fasta vcf=[variant_output].vcf shist=[stats].txt ploidy=1 rarity=0.45 usepairing=f border=0 qtrim=f trimq=0 realign=t repadding=70 rerows=3000 recols=3740 msa=MultiStateAligner9PacBio clearfilters minreadmapq=1 minreads=2 minqualitymax=10 minedistmax=20 minmapqmax=15 minstrandratio=0.1 minallelefraction=0.45**

6) Compress and index variant files (as for Illumina)

Tool: HTSlib

**bgzip [variant_output].vcf**

**tabix -p vcf [variant_output].vcf.gz**

7) Calculate variant statistics (as for Illumina)

Tool: VCFtools

**vcftools --gzvcf [compressed_and_indexed_variant_file].vcf.gz --out [output_name] ‑‑[option]**

note: exemplary options are e.g. --depth (mean depth per individual), --site-quality (per-site SNP quality), --SNPdensity 1000 (number and density of SNPs in bins of size 1000), --site-mean-depth (mean depth per site averaged across all individuals)

8) Create updated consensus (as for Illumina)

Tool: BCFtools

**bcftools consensus -f [reference_used_for_mapping].fasta -M N -o [output_consensus].fasta [compressed_and_indexed_variant_file].vcf.gz**

**F. Nanopore - *De novo* assembly**

1) *De novo* assembly

Tool: Canu

note: use fully processed reads for *de novo* assembly

**./canu -p [prefix_for_result_files] -d [/path/to/output/folder/] -nanopore-raw [fully_processed_reads].fasta genomeSize=0.18m minReadLength=300 minOverlapLength=300 correctedErrorRate=0.134 corOutCoverage=6000 readSamplingBias=2.0 readSamplingCoverage=400 corMhapSensitivity=low obtMhapSensitivity=low utgMhapSensitivity=low stopOnReadQuality=true stopOnLowCoverage=10 contigFilter="10 0 1.0 0.5 5"**

2) BLAST contigs to *Artemisia* to detect chimeric / contaminant contigs

Tool: BLAST+

note: if not already done, create a BLAST database of the *A. frigida* chloroplast with the IR_A_ removed, as described in D/5. Examine output.txt file visually regarding strand orientation of the hits and unmatched query regions (verify these via megaBLAST website searches)

**blastn -task megablast -query [Canu_contigs].fasta -db [name_of_database] -out [output_filename].txt -outfmt "7 qseqid qstart qend sstart send sstrand qcovhsp length pident bitscore evalue mismatch gapopen gaps"**

3) Split chimeric *Leucanthemum* contigs

Tool: *fastasubseq* (Exonerate package)

note: Exonerate uses an in-between coordinate system! Refer to manual at <https://www.ebi.ac.uk/about/vertebrate-genomics/software/exonerate-manual> for correct definition of subsequences. The example given below will separate a contig of 17,669 bp length into two sub-contigs of 11,014 bp and 6,655 bp, with the first number of the command line defining the start of the subsequence and the second giving its length

**./fastasubseq [contig_to_be_split].fasta 0 11014 > [name_of_first_contig].fasta**

**./fastasubseq [contig_to_be_split].fasta 11014 6655 > [name_of_second_contig].fasta**

4) Polishing of contigs

Tool: Nanopolish (requires Minimap2 and SAMtools)

note: processed Nanopore reads are first related to their original fast5 files (indexing), and are then mapped to the Canu contigs. Variants are extracted from the mapping after indexing, before improved contigs are generated. Variant calling is done separately for each contig, give the window (-w) in format "[contig_name]:0-[contig_length]", an example is given below

**./nanopolish index -s [path/to/Albacore/]sequencing_summary.txt -v -d [directory/containing/Albacore/fast5/files/] [processed_reads].fastq**

**./minimap2 -ax map-ont -t [desired_no_of_threads] [Canu_contigs].fasta [processed_reads].fastq | samtools sort -o [sorted_mapping_of_reads_to_contigs].bam -T [/path/to]/tmp**

**samtools index [sorted_mapping_of_reads_to_contigs].bam**

**./nanopolish variants --consensus -v -o variants_[contig_number_1-x].vcf -w "tig00000001:0-53891" --ploidy 1 -t [desired_no_of_threads] --max-haplotypes 30000 ‑‑min-candidate-frequency 0.2 --max-rounds 50 --calculate-all-support --reads [processed_reads].fastq --bam [sorted_indexed_mapping_of_reads_to_contigs].bam ‑‑genome [Canu_contigs].fasta**

**./nanopolish vcf2fasta -v -g [Canu_contigs].fasta variants_*.vcf > [polished_Canu_contigs].fasta**

5) Map polished contigs to *Artemisia* chloroplast

Tool: Minimap2, SAMtools

note: Based on the mapping, the correct order and starting positions of the contigs can be inferred using IGV. Contigs are then merged in BioEdit.

**./minimap2 -ax asm20 -t [desired_no_of_threads] [reference_A_frigida_without_IRa].fasta [polished_Canu_contigs].fasta > [mapped_contigs].sam**

**samtools view -b -h -o [mapped_contigs].bam [mapped_contigs].sam**

**samtools sort -o [mapped_contigs_sorted].bam [mapped_contigs].bam**

**samtools index -b [mapped_contigs_sorted].bam [mapped_contigs_sorted].bai**

6) Obtain read coverage of *de novo* assembly via mapping input reads

Tool: *mapPacBio.sh* (BBTools software suite)

note: use the reads actually used for assembly by Canu (after correction and trimming steps), named [prefix].trimmedReads.fasta

**gunzip [prefix].trimmedReads.fasta.gz**

**./mapPacBio.sh ref=[de_novo_assembled_fasta_without_IRa].fasta nodisk=t in=[prefix].trimmedReads.fasta out=[coveragemapping].sam maxlen=3000 vslow=t maxindel=100 threads=[desired_no_of_threads] ambiguous=random mappedonly=t scafstats=[scafstats].txt sortscafs=t statsfile=[stats].txt covstats=[covstats].txt basecov=[basecov].txt secondarycov=f**

**G. Nanopore/Illumina - Hybrid *de novo* assembly using Unicycler**

1) *De novo* assembly

Tool: Unicycler

note: use processed Illumina reads and fully processed Nanopore reads; provide racon path only if the program throws a respective error

**unicycler -1 [Illumina_processed_reads_1].fastq -2 [Illumina_processed_reads_2].fastq -l [Nanopore_fully_processed_reads].fasta -o [path/to/output/folder/] -t [desired_no_of_threads] --mode conservative --racon_path [path/to/bin/racon]**

**H. Nanopore/Illumina - Hybrid *de novo* assembly using Nanocorr**

1) *De novo* assembly

Tool: (Nanocorr, see D/7), Canu

note: use fully processed, improved Nanopore reads (from D/7)

**./canu -p [prefix_for_result_files] -d [/path/to/output/folder/] -nanopore-raw [Nanopore_fully_processed_reads_improved].fasta genomeSize=0.18m minReadLength=2000 minOverlapLength=300 correctedErrorRate=0.134 corOutCoverage=6000 readSamplingBias=0.0 readSamplingCoverage=200 corMhapSensitivity=low obtMhapSensitivity=low utgMhapSensitivity=low stopOnReadQuality=false stopOnLowCoverage=10 contigFilter="10 0 1.0 0.5 5"**

2) BLAST contigs to *Artemisia* to detect chimeric / contaminant contigs
 (as for Nanopore unimproved *de novo* assembly)

Tool: BLAST+

note: if not already done, create a BLAST database of the *A. frigida* chloroplast with the IR_A_ removed, as described in D/5. Examine output.txt file visually regarding strand orientation of the hits and unmatched query regions (verify these via megaBLAST website searches)

**blastn -task megablast -query [Canu_contigs].fasta -db [name_of_database] -out [output_filename].txt -outfmt "7 qseqid qstart qend sstart send sstrand qcovhsp length pident bitscore evalue mismatch gapopen gaps"**

3) Split chimeric *Leucanthemum* contigs

(as for Nanopore unimproved *de novo* assembly)

Tool: *fastasubseq* (Exonerate package)

note: Exonerate uses an in-between coordinate system! Refer to manual at <https://www.ebi.ac.uk/about/vertebrate-genomics/software/exonerate-manual> for correct definition of subsequences. The example given below will separate a contig of 17,669 bp length into two sub-contigs of 11,014 bp and 6,655 bp, with the first number of the command line defining the start of the subsequence and the second giving its length

**./fastasubseq [contig_to_be_split].fasta 0 11014 > [name_of_first_subcontig].fasta**

**./fastasubseq [contig_to_be_split].fasta 11014 6655 > [name_of_second_subcontig].fasta**

note: Do NOT polish contigs as in F/4 after this step as this might deteriorate them!

4) Map contigs to *Artemisia* chloroplast

(as for Nanopore unimproved *de novo* assembly)

Tool: Minimap2, SAMtools

note: Based on the mapping, the correct order and starting positions of the contigs can be inferred using IGV. Contigs are then merged in BioEdit.

**./minimap2 -ax asm20 -t [desired_no_of_threads] [reference_A_frigida_without_IRa].fasta [Canu_contigs].fasta > [mapped_contigs].sam**

**samtools view -b -h -o [mapped_contigs].bam [mapped_contigs].sam**

**samtools sort -o [mapped_contigs_sorted].bam [mapped_contigs].bam**

**samtools index -b [mapped_contigs_sorted].bam [mapped_contigs_sorted].bai**

5) Obtain read coverage of *de novo* assembly via mapping input reads

(as for Nanopore unimproved *de novo* assembly)

Tool: *mapPacBio.sh* (BBTools software suite)

note: use the reads actually used for assembly by Canu (after correction and trimming steps), named [prefix].trimmedReads.fasta

**gunzip [prefix].trimmedReads.fasta.gz**

**./mapPacBio.sh ref=[de_novo_assembled_fasta_without_IRa].fasta nodisk=t in=[prefix].trimmedReads.fasta out=[coveragemapping].sam maxlen=3000 vslow=t maxindel=100 threads=[desired_no_of_threads] ambiguous=random mappedonly=t scafstats=[scafstats].txt sortscafs=t statsfile=[stats].txt covstats=[covstats].txt basecov=[basecov].txt secondarycov=f**

**I. Sequence variation between *L. vulgare* and *L. virgatum***

1) Mapping (as for Illumina reference-based mapping assembly)

Tool: BBMap (BBTools software suite)

note: Illumina data are used for this step; map processed *L. virgatum* reads to the *L. vulgare de novo* assembly (IR_A_ removed)

**./bbmap.sh ref=[de_novo_assembled_L_vulgare_without_IRa].fasta nodisk=t in1=[L_virgatum_processed_reads_1].fastq in2=[L_virgatum_processed_reads_2].fastq out=[mapped_reads].sam vslow=t maxindel=300 threads=[desired_no_of_threads] ambiguous=random killbadpairs=t pairedonly=t pairlen=180 mappedonly=t showprogress2=10 mdtag=t nhtag=t nmtag=t stoptag=t lengthtag=t idtag=t inserttag=t boundstag=t scafstats=[scafstats].txt sortscafs=t qhist=[qhist].txt lhist=[lhist].txt ihist=[ihist].txt ehist=[ehist].txt indelhist=[indelhist].txt statsfile=[stats].txt covstats=[covstats].txt basecov=[basecov].txt secondarycov=f**

2) Convert (.sam to .bam), sort and index mappings

(as for Illumina reference-based mapping assembly)

Tool: SAMtools

**samtools view -b -h -o [mapped_reads].bam [mapped_reads].sam**

**samtools sort -o [mapping_sorted].bam [mapped reads].bam**

**samtools index -b [mapping_sorted].bam [mapping_sorted].bai**

3) Remove duplicate reads from L. virgatum and Artemisia mappings

(as for Illumina reference-based mapping assembly)

Tool: Picard tools

**picard MarkDuplicates Remove_Duplicates=true I=[mapping_sorted].bam O=[mapping_sorted_nodup].bam M=[log].txt**

4) Variant calling

(as for Illumina reference-based mapping assembly)

Tool: *callvariants2.sh* (BBTools software suite)

**./callvariants2.sh in=[mapping_sorted_nodup].bam ref=[de_novo_assembled_L_vulgare_without_IRa].fasta vcf=[variant_output].vcf shist=[stats].txt ploidy=1 rarity=0.51 border=0 qtrim=f trimq=0 realign=f minreadmapq=1 minallelefraction=0.51**

5) Compress and index variant files

(as for Illumina reference-based mapping assembly)

Tool: HTSlib

**bgzip [variant_output].vcf**

**tabix -p vcf [variant_output].vcf.gz**

6) Calculate number and density of SNPs in 1000-bp windows

Tool: VCFtools

**vcftools --gzvcf [compressed_and_indexed_variant_file].vcf.gz --out [output_name] ‑‑SNPdensity 1000**
