# Supplementary figures and images for: Can we use it? On the utility of de novo and reference-based assembly of Nanopore data for plant plastome sequencing
Source: PLoS One. 2020 Mar 24;15(3):e0226234. doi: 10.1371/journal.pone.0226234 (PMC7092973; doi:10.1371/journal.pone.0226234)

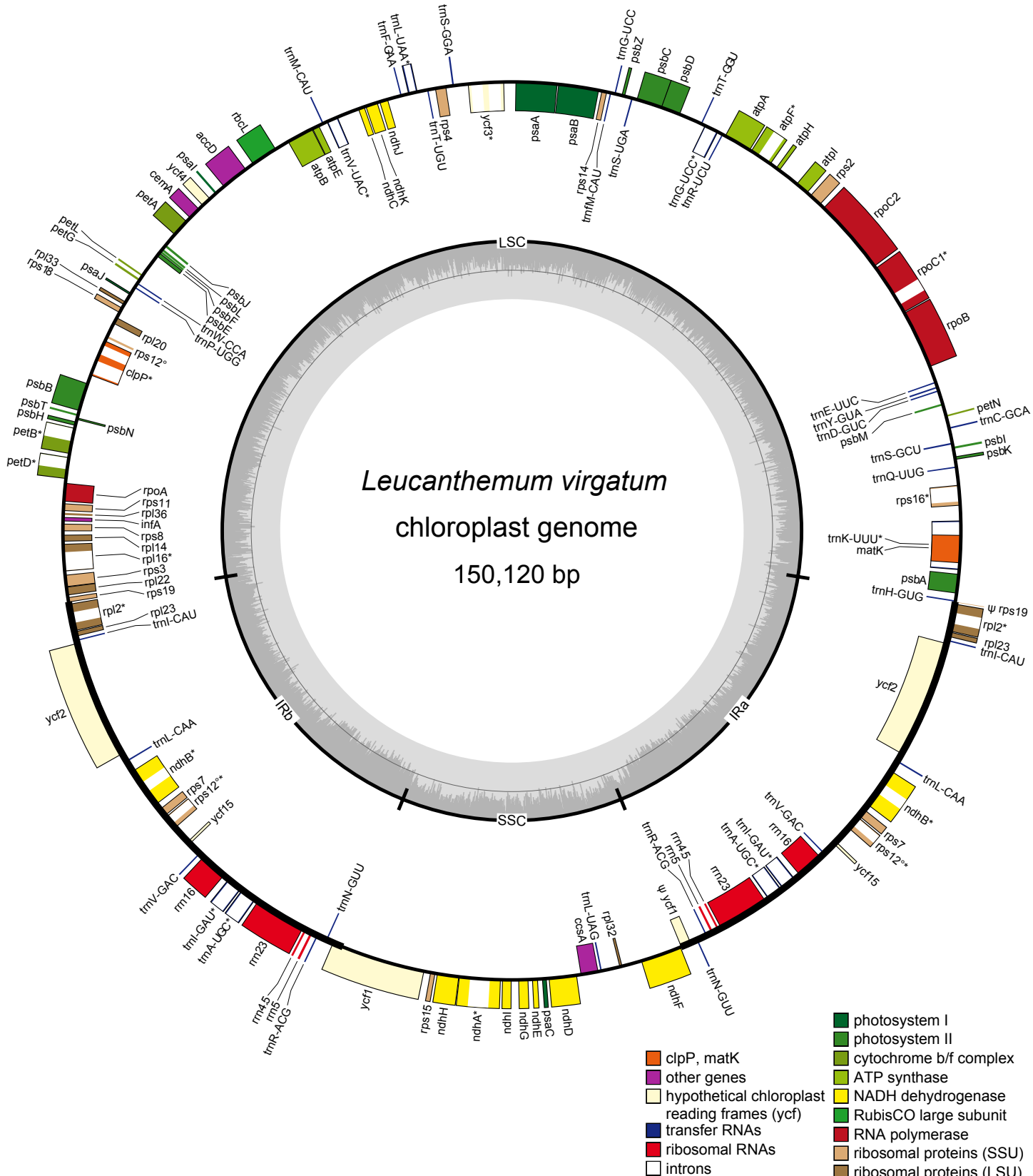

Supplement: S1 Fig — Genes on the outside of the outer circle are transcribed counterclockwise, genes on the inside are transcribed clockwise. Introns are illustrated with white color within genes; genes containing an intron are additionally marked with *. Pseudogenes are preceded by a ψ. The trans-spliced rps12 gene is marked with °. Color-coding of genes depicts their affiliation to the functional groups given. The inner circle indicates the borders of the large single-copy (LSC) and small single-copy (SSC) regions as well as the inverted repeats (IR). The innermost gray shaded area shows the G+C content of the cp genome. The gene order is identical in L. vulgare (see Fig 2), whereas their exact positions and the extent of the inverted repeat slightly differ (Fig 3). (PDF) [file pone.0226234.s006.pdf]
